# Acceptance and commitment therapy for patients with chronic pain: A systematic review and meta-analysis on psychological outcomes and quality of life

**DOI:** 10.1371/journal.pone.0301226

**Published:** 2024-06-14

**Authors:** Lu Ye, Yunhua Li, Qingchuan Deng, Xin Zhao, Lili Zhong, Li Yang

**Affiliations:** 1 Department of Oncology, The Second Affiliated Hospital of Chengdu Medical College, China National Nuclear Corporation 416 Hospital, Chengdu, China; 2 College of Education, Chengdu College of Arts and Sciences, Chengdu, Sichuan, China; 3 School of Nursing, Sichuan Nursing Vocational College, Chengdu, Sichuan, China; 4 Department of Nephrology, West China Hospital of Sichuan University, Chengdu, Sichuan, China; Monash University Malaysia, MALAYSIA

## Abstract

**Objectives:**

To assess the efficacy of acceptance and commitment therapy (ACT) for patients with chronic pain.

**Materials and methods:**

The research conducted a systematic search of the Cochrane Library, Web of Science, PubMed, EMBASE, PsycINFO, and Cumulative Index of Nursing and Allied Health Literature (CINAHL) databases following the PRISMA guidelines. The retrieval time limit was from the establishment of the database to October 2023. A meta-analysis was carried out for the randomized controlled trials (RCTs) that meet the inclusion and exclusion criteria by using RevMan 5.3.

**Results:**

Twenty-one RCTs were included. At post-treatment, a significant medium effect size (ES) was found in measuring pain interference, functional impairment, pain acceptance, psychological inflexibility, and depression; Pain intensity, anxiety, and quality of life (QOL) had a small ES. At three months post-treatment, a large ES was found in measuring functional impairment, and a medium ES was found in the other indicators.

**Conclusion:**

The researchers provided evidence for the effectiveness of ACT as an intervention for patients with chronic pain, which can be applied by clinicians or nurses in practice. Future research should explore the applicability of ACT to different pain conditions and modalities.

**Implications for nursing:**

Post-treatment data highlight the efficacy of ACT in moderating pain-related outcomes. Clinical nurses are encouraged to incorporate ACT into routine patient education and interventions, including promoting pain acceptance, promoting mindfulness practices, and using cognitive stress reduction techniques. Standardized follow-up after an ACT intervention for patients with chronic pain is critical, including regular assessment, feedback, and realignment of treatment strategies. Overall, ACT became an important tool for nurses to improve the lives of patients with chronic pain.

## Introduction

Chronic pain is a prevalent and debilitating health issue worldwide. It refers to pain that persists beyond three months and is associated with significant emotional distress or functional impairment, such as anxiety, depression, cognitive impairment, and reduced physical activity [1]. A national health interview survey in the United States revealed that about 50.2 million adults (20.5%) experienced pain on most or every day [2]. The prevalence of chronic pain was slightly higher in the European Union, at about 27% [3]. Chronic pain not only affects a large proportion of the population, but it also imposes many restrictions on the individual [2]. Chronic pain impairs work performance and increases absenteeism [4–6]. Chronic pain also hinders daily functioning and social participation, as one study found that 40% of individuals with chronic pain had difficulty walking, and 34% had difficulty socializing [7, 8]. Moreover, chronic pain is linked to depression, anxiety, and lower quality of life (QOL) [9–11]. In general, chronic pain is one of the most common health challenges globally, with significant direct or indirect adverse consequences for patients, their families, and society [5, 12, 13].

Psychological interventions are essential for chronic pain management [11, 14]. Research indicates that attempts to control pain often result in increased pain severity [15, 16]. For instance, efforts to manage pain may trigger additional psychological stress, leading to elevated levels of stress hormones, thereby amplifying the perception of pain [17]. Moreover, excessive focus on pain can lead to an attentional focusing effect, consequently heightening individuals’ sensitivity to painful stimuli [18]. Conversely, when patients attempt to accept pain, their pain intensity decreases, and their mental health improves, which is one of the aims of Acceptance and Commitment Therapy (ACT) [19, 20]. ACT is a contextually focused form of cognitive behavioral psychotherapy that uses mindfulness and behavioral activation to increase patients’ psychological flexibility [20, 21]. It helps patients engage in values-based, positive behaviors while experiencing difficult thoughts, emotions, or sensations [21, 22]. ACT has six core processes: acceptance, being present, cognitive defusion, self as context, values, and committed action [20]. Unlike traditional cognitive behavior therapy (CBT), ACT encourages individuals to accept and confront their pain [20]. Based on this, individuals are motivated to discover their self-worth and pursue positive actions aligned with their values in order to achieve their life goals and values [16, 23, 24]. Therefore, theoretically, ACT can be an effective alternative treatment for patients with chronic pain to enhance their functioning and reduce the impact of chronic pain [15].

Our literature review shows that multiple systematic reviews have confirmed that clinical personnel using ACT can effectively improve certain indicators of patients with chronic pain, such as anxiety, depression, and pain acceptance [23, 25, 26]. For instance, a systematic review conducted by Hughes and colleagues, encompassing 11 trials, demonstrated that ACT yielded significant moderate to large effects in terms of pain acceptance and psychological flexibility, along with small to moderate effects in functionality, anxiety, and depression, but no significant improvements were observed in QOL [25]. In another systematic review by Du and associates, which focused on the efficacy of ACT in improving functioning in individuals with chronic pain, 12 randomized controlled trials (RCTs) were included [27]. This review found that ACT had an immediate positive impact on the functioning of patients with chronic pain, yet the long-term effects remain unclear [27]. Inês and colleagues focused on technology-based ACT interventions for patients with chronic pain, incorporating 5 RCTs with a total of 746 participants [26]. They observed that, compared to control groups, technology-based ACT demonstrated moderate effects in pain intervention and pain acceptance, and smaller effects in depression, mindfulness, and psychological flexibility [26].

However, it has been six years since the systematic review by Hughes and colleagues, which focused on the comprehensive psychological outcomes of chronic pain patients treated with various forms of ACT by clinical practitioners in RCTs [25]. Since then, numerous new high-quality RCTs on this topic have emerged. These RCTs have made many improvements in research design, refinement of pain types in subjects, and standardization of ACT interventions. For example, Hansen’s 2023 study demonstrated advancements in design, participant engagement, and intervention customization, especially by having endometriosis patient complete homework for 10 weeks, using anonymous IDs to ensure blinding, and employing a 12-week pain diary to improve data quality [28]. Moreover, by combining Mindfulness-Based Stress Reduction and ACT in the tailored intervention, the research not only enhanced the intervention’s relevance and effectiveness but also offered valuable methodological insights for future ACT research in chronic pain management [28]. Lina’s 2022 research used an automated web program for individual-level crossover block randomization to ensure fair allocation across study arms [29]. A blinded third party unconnected to the research conducted the randomization and allocation, while data collectors were kept unaware of participant groups [29]. Despite the inability to blind participants, the study effectively minimized bias and optimized follow-up through regular online assessments and detailed flowcharts. In general, including these new high-quality studies will also improve the accuracy of our systematic review. In addition, according to Cochrane’s recommendations, if a systematic review on this topic exceeds a certain number of years based on its academic value and practical value, it is also necessary to update it [30, 31].

Secondly, many new and effective pain management concepts have emerged in the field of pain management during these six years, such as emphasizing the importance of pain interference indicators [32]. However, these indicators were missing from Hughes and his colleagues’ systematic review [25]. Therefore, in this systematic review we have also included pain interference and other indicators as our outcome indicators so that the intervention effects of ACT can match with the latest pain concepts.

Finally, we have also conducted a subgroup analysis in this systematic review. The grouping criterion is the time when the outcome indicators were measured after the participants received ACT intervention. We have divided our analysis into two subgroups: post-treatment and three months post-treatment hoping to improve our research quality through this subgroup analysis research design and reduce bias in research results.

### Research problem and aim

The primary objective and problem addressed by this study is to conduct an extensive systematic review and meta-analysis to evaluate the efficacy of ACT in patients with chronic pain, particularly in light of recent research advancements and evolving concepts in pain management. This will encompass:

1. Analyzing all new RCTs on ACT interventions in chronic pain, published in English academic journals, especially those within the last six years, and incorporating them into our systematic review.
2. Investigating novel pain intervention indicators, such as pain interference, in the context of ACT interventions in chronic pain patients, to provide evidence-based recommendations for pain management guidelines.
3. Conducting subgroup analyses with a special focus on the effects at different time points post-ACT intervention (immediately after treatment and three months post-treatment). This will aid in understanding the short-term and long-term effects of ACT on chronic pain patients, the sustainability of these effects, and in formulating effective intervention strategies.

## Materials and methods

This systematic review was guided by the preferred reporting items for systematic review and meta-analysis protocols (PRISMA) recommendations [33].

### Search strategy

From inception to October 1, 2023, the systematic review systematically searched six databases: the Cochrane Library, Web of Science, PubMed, EMBASE, PsycINFO, and CINAHL. In addition, peer-reviewed manuscripts published in English were included within the systematic review. The search term was a combination of medical subject headings (MeSH) terms along with free terms and was adjusted according to the specific database, mainly including “acceptance and commitment therapy” AND “chronic pain” AND “randomized controlled trial.” S1 Appendix presents the search strategies utilized for each database.

### Inclusion and exclusion criteria

Studies were eligible for inclusion if they met the following PICOS elements:

**(1) P (Population)**:

Participants were ≥16 years of age and suffered from chronic pain lasting more than three months and beyond [34]. The type of pain was not limited, but the pain associated with malignancy was excluded [35].

**(2) I (Intervention)**:

The intervention of qualified studies should be ACT that explicitly includes both acceptance and commitment components [25]; studies of the intervention focusing on only one of these components would be excluded. Therefore, studies based on mindfulness were not included.

**(3) C (Control)**:

The intervention in the control group was treatment as usual (TAU) or waiting list (WL). When the interventions in the control group were other interventions such as relaxation therapy, CBT, or exercise, they were excluded.

**(4) O (Outcome)**:

The primary purpose of ACT is to help individuals live more productive and meaningful lives and improve their functional status, not to control symptoms [23]. Therefore, the systematic review used pain interference and functional impairment as the primary outcomes in this study. Pain intensity, pain acceptance, psychological inflexibility, anxiety, depression, and QOL were used as secondary outcome measures.

**(5) S (Study Design):** RCTs.

### Screening procedure

The researchers used EndNote X9 software to manage the database search results. The first step was to remove duplicate studies using the software. In this study, two independent reviewers (YH and QC) completed the screening, review, and data extraction for studies included in the research. As a preliminary trial, both reviewers independently reviewed 50 papers. During this process, YH included 46 articles, while QC included 45. They reached a consensus on including 45 papers and unanimously excluded 4. This high level of agreement is reflected in a Kappa value of approximately 0.88, indicating a strong consistency in the application of screening criteria between the two assessors. This result confirms the reliability and effectiveness of our screening process. Then, the two independent reviewers (YH and QC) continued to assess the titles, abstracts, and keywords of the remaining studies using predefined inclusion and exclusion criteria, eliminating those that did not meet the standards. Subsequently, these reviewers carefully read the full texts of the studies that passed the initial screening, further excluding any that failed to meet the established criteria. This process resulted in the final selection of studies. Throughout this review process, each reviewer worked independently, and any disagreements were discussed collectively. If a consensus could not be reached, a third reviewer (LY) was consulted to resolve the disputed decisions.

### Data extraction

The researchers used a prepiloted, standardized form to extract information for each experiment. Information extracted included study characteristics (author, country, year of publication), participant information (age, gender, pain type, pain duration), intervention details (mode of intervention, intervention duration, treatment of the control group), and outcome measures. During this process, if missing data are identified, necessary conversions of the data was conducted or the authors were directly contacted to acquire the data, in order to ensure the quality of the systematic review results. Throughout this stage, the extraction of information was conducted independently by two reviewers (YH and QC). If there was a disagreement in the extracted information, the disagreement was resolved by discussion or a third reviewer (LY).

### Risk of bias: Quality assessment

The researchers used the Cochrane risk of bias tool [36] and part one of the Yates quality rating scale [37] to assess the methodological quality of the included studies. Two reviewers (YH and QC) independently evaluated the included studies. Discussions or a third reviewer (LY) resolved any discrepancies in the evaluation.

The Cochrane Assessment of Bias tool [36] was used to assess the methodological quality and to capture the risk of bias in RCTs [36]. Each item was assessed as having low, unclear, or high risk of bias. Items were not summed to obtain a total score, as recommended by the Centre for Reviews and Dissemination CRD [38]. The Cochrane risk of bias tool [36] evaluated the included studies in the following seven areas: random sequence generation (selection bias), allocation concealment (selection bias), blinding of participants and personnel (performance bias), blinding of outcome assessment (detection bias), incomplete outcome data (attrition bias), selective reporting (reporting bias), other bias.

Part one of the Yates quality rating scale [37] is a tool used to assess specific sources of bias for psychological RCTs that the Cochrane risk of bias tool [36] failed to capture. Items were scored (1 or 2 points) according to whether the construct was reported. The tool evaluated the included studies in the following six areas: rationale, treatment duration, treatment manualization, adherence to the manual, therapist training, and client engagement.

### Statistical analysis and meta-analysis

The researchers used RevMan 5.3 software to conduct a meta-analysis, generate forest plots, and calculate statistics for all data. RevMan is software provided by the Cochrane Collaboration for systematic reviewers. It is mainly used to create and save the protocol or full text of Cochrane systematic reviews, perform meta-analysis on the input data, and display the meta-analysis results in more intuitive formats, such as forest plots, and to update systematic reviews [39].

The systematic review used chi-square tests to determine if heterogeneity existed between studies. If P > 0.1, and I^2^ < 50%, the study was not statistically heterogeneous; if P < 0.1, and I^2^ > 50%, the study was statistically heterogeneous. When heterogeneity was significant, sensitivity analysis or subgroup analysis was used to determine the source of heterogeneity. To address the heterogeneity detected, we not only explored its potential sources, such as through subgroup analyses, but also implemented sensitivity analyses to test the robustness of our results. The sensitivity analyses involved switching between random-effects and fixed-effects models to assess the impact of different effect models on the estimation of Effect Sizes (ES). Furthermore, we investigated the stability of the results by sequentially excluding each study, a process that helped us ascertain that no single study exerted undue influence on the overall outcomes. In conducting subgroup analyses, we focused on evaluating the effects of ACT interventions on patients with chronic pain at two critical time points: the end of treatment and three months post-treatment. The selection of these time points was grounded in the emphasis on short-term versus long-term effects noted in prior research, as well as the importance of understanding the persistence of intervention effects. We anticipated that the various outcome measures in patients with chronic pain might exhibit different effects immediately post-treatment and at three months post-treatment. Such variations could reveal insights into the durability and evolving trends of ACT interventions, thereby providing a basis for clinicians to develop intervention strategies.

For continuous data, weighted mean differences (WMDs) with 95% confidence interval (CI) were used if each study used the same measurement tool to measure the same outcome indicator. Conversely, standardized mean differences (SMDs) with 95% CI were used. For the purpose of this study, the random effects model was selected within the systematic review to calculate ES [40]. According to the viewpoint of Cohen, ES < 0.2 is considered a negligible effect, 0.2 ≤ ES < 0.5 is small, 0.5 ≤ ES < 0.8 is medium, and ES ≥ 0.8 is large [41]. At the same time, the systematic review used RevMan 5.3 to draw a funnel plot to measure its publication bias.

## Result

### Search results

**Fig 1:**
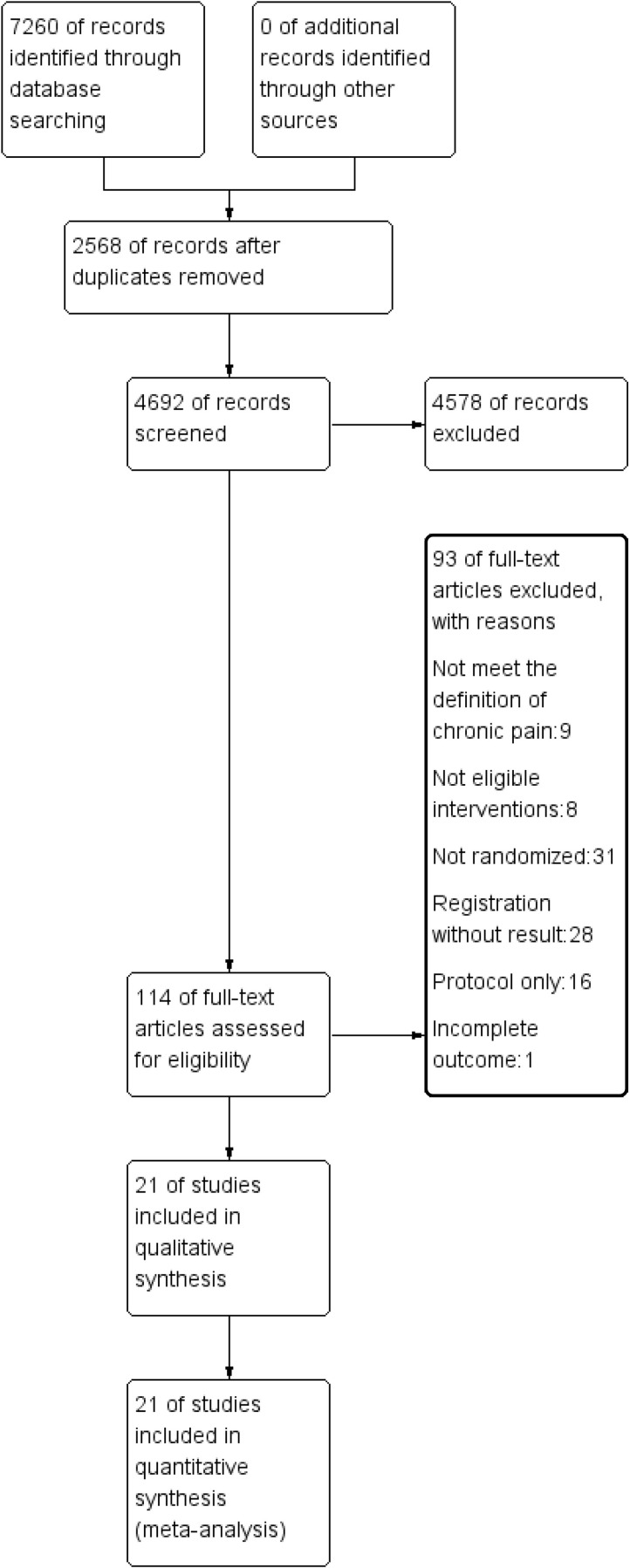


A searching of the databases obtained 7260 articles (Fig 1). 2568 duplicates were excluded, 4578 irrelevant articles were excluded after reading the titles and abstracts, and 114 articles were obtained after the initial screening. 93 articles were excluded after reviewing the full text, and 21 articles were finally included [28, 29, 42–60]. The selection of the studies is summarized in the PRISMA diagram in Fig 1.

### Characteristics of selected studies

Table 1 presents the characteristics of the selected studies. Of the 21 studies included, 16 were from Europe, 3 from North America, and 2 from Oceania. The overall sample size was 1298, with a mean age ranging from 29.5 to 82.26, and the majority were female. In addition, three studies reported subjects with a specific type of chronic pain, Fibromyalgia [49, 51, 58]. Thirteen studies reported an average chronic pain duration for the sample, ranging from 6.8 to 25.34 years. The forms of ACT intervention included in the study were face-to-face individual intervention, face-to-face group intervention, online intervention, and self-help intervention. Control group interventions were either TAU or WL.

**Table 1:** Characteristics of included studies (K = 21).

STUDY (COUNTRY)	N (FEMALE,%)	MEAN AGE (SD)(y)	Type of pain PAIN DURATION (M [SD])(y)	INTERVENTION(S)	COMPARATOR(S)	OUTCOME MEASURES	QUALITY SCORE (YATES QUALITY ASSESSMENT TOOL
Alonso-Fernández et al. (2016), Spain	53 (78.1)	Total: 82.26(10.00)	Chronic pain; Intervention: 21.30(20.81) Comparator: 25.34(20.36)	Therapist-delivered ACT group; Nine 120-min weekly group sessions	TAU + a 2h educational group session	Pain interference: BPI Pain intensity: BPI Pain acceptance: CPAQ Anxiety: PASS Depression: GDS	7
Andrea et al. (2022), Sweden	88 (100)	Intervention: 24.2(5.2) Comparator: 24.7(3.3)	Vulvodynia; Intervention: 5.2(4.7) Comparator: 4.6(3.5)	Guided Internet-delivered ACT; 6-section weekly program	TAU	Pain intensity: NRS Pain acceptance: CPAQ	8
Buhrman et al. (2013), Sweden	76 (59.2)	Intervention: 48.8(9.48) Comparator: 49.3(11.26)	Chronic pain; Intervention: 13.1(10.58) Comparator: 17.4(12.38)	Guided Internet-delivered ACT; 7-section weekly program	WL	Pain intensity: MPI Pain acceptance: CPAQ Anxiety: HADS Depression: HADS Quality of life: QOLI	7
Dahl et al. (2004), Sweden	19 (89.5)	Intervention: 36.7(12.5) Comparator: 44.4(13.6)	Chronic pain; Intervention: 7.0(6.4) Comparator: 7.0(10.4)	Therapist-delivered individual ACT; 4*60 min individual Sessions Weekly + medical TAU	Medical TAU	Pain intensity: NRS Quality of life: LSQ	6
Hansen et al. (2023), Denmark	35 (100)	Intervention: 28.95(7.84) Comparator: 32.81(9.01)	persistent pain in Endometriosis; Intervention: 14.00(8.28) Comparator: 12.93(7.51)	The self-help book + Therapist-delivered ACT group; 10*180 min group Sessions;	WL	Pain intensity: NRS Pain acceptance: CPAQ	8
Johnston et al. (2010), New Zealand	14 (62.5)	Total: 43 (/)	Chronic pain; Pain duration: /	The self-help ACT book + weekly telephone support	WL	Pain intensity: SFMPQ Pain acceptance: CPAQ Anxiety: BAI Depression: CMDI Quality of life: QOLI	6
Josée et al. (2019), Canada	130 (81.5)	Intervention: 51.9(14.2) Comparator: 50.2(11.0)	Chronic pain; Pain duration: /	The self-help ACT email + weekly telephone support	WL	Pain acceptance: CPAQ Depression: BDI Psychological Inflexibility: PIPS	7
Kanzler et al. (2022), USA	23 (53.8)	Total: 52 (/)	Non-cancer chronic pain; Intervention: 9.72(7.38) Comparator: 14.03(13.07)	Guided delivered ACT; A 30-min individual focused ACT visit + three 1h weekly group visits + a “booster” visit	TAU	Pain acceptance: CPAQ Functional impairment: ODI	6
Lin et al. (2017), Germany	161 (85.5)	Intervention: 51.7(12.3) Comparator: 50.3(12.5)	Chronic pain; Intervention: 9.66(11.37) Comparator: 7.91(8.21)	Guided Internet delivered ACT; eight 60-min modules	WL	Functional impairment: BPI Pain interference: MPI Pain intensity: NUM Pain acceptance: CPAQ Psychological Inflexibility: AAQ-II Anxiety: GAD	7
Lina et al. (2022), Germany	57 (70.4)	Intervention: 57.23(9.50) Comparator: 56.68(7.70)	Chronic Pain Pain duration: /	Guided Internet-delivered ACT; 7-section weekly program	TAU + A piece of psychological education material	Pain intensity: NRS Pain interference: MPI Pain acceptance: CPAQ Depression: QIDS Quality of life: AQoL	8
Luciano et al. (2014), Spain	96 (96.2)	Intervention: 48.88(5.94) Comparator: 48.28(5.71)	Fibromyalgia; Intervention: 14.08(8.96) Comparator: 12.98(8.74)	Therapist-delivered ACT group; 8*150 min group sessions	WL	Functional impairment: RIQ Pain acceptance: CPAQ Anxiety: HADS Depression: HADS	7
Martin et al. (2021), USA	64 (61.3)	Intervention: 31.8(11.9) Comparator: 29.5(11.2)	Chronic pain; Pain duration: /	Guided delivered ACT; Two, 2-h in-person sessions + eight weeks of online training	WL	Pain interference: PPIS Pain intensity: NRS Pain acceptance: CPAQ Anxiety: PASS	7
McCracken et al. (2013), England	58 (68.5)	Total: 58.0(12.8)	Chronic pain; Pain duration: /	Therapist-delivered ACT group; 4*240 min group sessions	WL	Functional impairment: RMDQ Pain acceptance: CPAQ Depression: PHQ	7
Rickardsson et al. (2021), Sweden	79 (91.1)	Intervention: 48.4(13.1) Comparator: 50.6(11.1)	Chronic pain; Intervention: 18.9(12.7) Comparator: 17.3(13.6)	Guided Internet delivered ACT; 7 modules over 8 weeks	WL	Pain interference: PII Pain intensity: NRS Psychological Inflexibility: PIPS Anxiety: GAD Depression: PHQ Quality of life: EQ-5D	7
Scott et al. (2018), British	48 (63.4)	Intervention: 47.26(14.0) Comparator: 43.84(13.9)	Chronic pain; Pain duration: /	Guided Internet-delivered ACT; 8 sessions twice weekly	TAU	Functional impairment: WSAS Pain intensity: NRS Pain acceptance: CPAQ Psychological Inflexibility: PIPS Depression: PHQ	7
Scott et al. (2021), UK	31 (23.7)	Total: 55.87(5.77)	HIV-infected patients with chronic pain; Pain duration: 11.44 (8.16)	Guided online delivered ACT; 12 online sessions (45–60 min each) over 8 weeks	WL	Functional impairment: WSAS Pain intensity: BPI Pain acceptance: CPAQ Depression: PHQ	8
Simister et al. (2018), Canada	58 (/)	Total: 39.7(9.36)	Fibromyalgia; Pain duration: /	Video-based self-help ACT; 7 modules over 2 months	TAU	Pain acceptance: CPAQ Depression: CES-D	7
Trompetter et al. (2015), Holland	121 (76)	Intervention: 52.9(13.3) Comparator: 53.2(12.0)	Chronic Pain; Pain duration: /	Guided Internet delivered ACT; 9 modules over 9–12 weeks	WL	Pain interference: MPI Pain intensity: NRS Psychological inflexibility: PIPS Anxiety: HADS Depression: HADS	8
Vasiliou et al. (2020), Ireland	62 (84)	Total: 42.89(10.27)	Chronic Pain; Pain duration: 18.09(10.71)	Therapist-delivered ACT group; The 8-weekly, 1.5-hour treatment sessions	WL	Pain acceptance: CPAQ Pain intensity: BPI Pain acceptance: CPAQ Functional impairment: HDI Anxiety: HADS Depression: HADS	7
Wicksell et al. (2008), Sweden	19 (76.2)	Intervention: 48.2(7.8) Comparator: 55.1(11.2)	Chronic pain; Intervention: 7.0(3.5) Comparator: 6.8(3.5)	Therapist-delivered individual ACT; 10*60 min sessions, over 8 weeks	WL	Functional impairment: PDI Pain interference: 10-cm VAS Pain intensity:10-cm VAS Psychological inflexibility: PIPS Anxiety: HADS Depression: HADS Quality of life: SLS	8
Wicksell et al. (2013) Sweden	36 (100)	Total: 45.1(6.6)	Fibromyalgia; Pain duration: 11.8(7.2)	Therapist-delivered ACT group; 12 weekly group ACT sessions (90 min each)	WL	Functional impairment: PDI Pain intensity: NRS Pain acceptance: CPAQ Psychological inflexibility: PIPS Anxiety: STAI Depression: BDI Quality of life: SF-36	8

**Abbreviations:** AAQ-II: the German version of the Acceptance and Action Questionnaire-II; AQoL: The Assessment of Quality of Life; BAI: Beck Anxiety Inventory; BDI: Beck Depression Inventory; CES-D: The Centre for Epidemiological Studies Depression Scale; CPAQ: Chronic Pain Acceptance Questionnaire; CMDI: Chicago Multi-scale Depression Inventory; EHP: The Endometriosis Health Profile; EQ-5D: The European Quality of Life; GAD: Generalized Anxiety Disorder Screen; GDS: the Geriatric Depression Scale; HADS: Hospital And Anxiety Depression Scale; HDI: The Henry Ford Hospital Headache Disability Inventory; LSQ: Life Satisfaction Questionnaire; MPI: Multidimensional Pain Inventory; NRS: The Numeric Rating Scale; ODI: Oswestry Disability Index; PASS-20: The Pain Anxiety Symptoms Scale; PDI: Pain Disability Index; PHQ: the Patient Health Questionnaire; PIPS: The Psychological Inflexibility in Pain Scale; PII: the Pain Interference Index; PPIS: the PROMIS Pain Interference short form; QOLI: Quality Of Life Inventory; QIDS: The Quick Inventory of Depressive Symptomatology; RIQ: Romyalgia Impact Questionnaire; RMDQ: the Roland and Morris Disability Questionnaire; SF-36: The Short Form-36 Health Survey; SFMPQ: Short-Form McGill Pain Questionnaire; SLS: Satisfaction with Life Scale; STAI: The Spielberger Trait-State Anxiety Inventory; VAS: Visual Analogue Scales; WSAS: The WSAS-Work and Social Adjustment Scale

### Quality assessment

#### The Cochrane risk of bias tool

**Fig 2:**
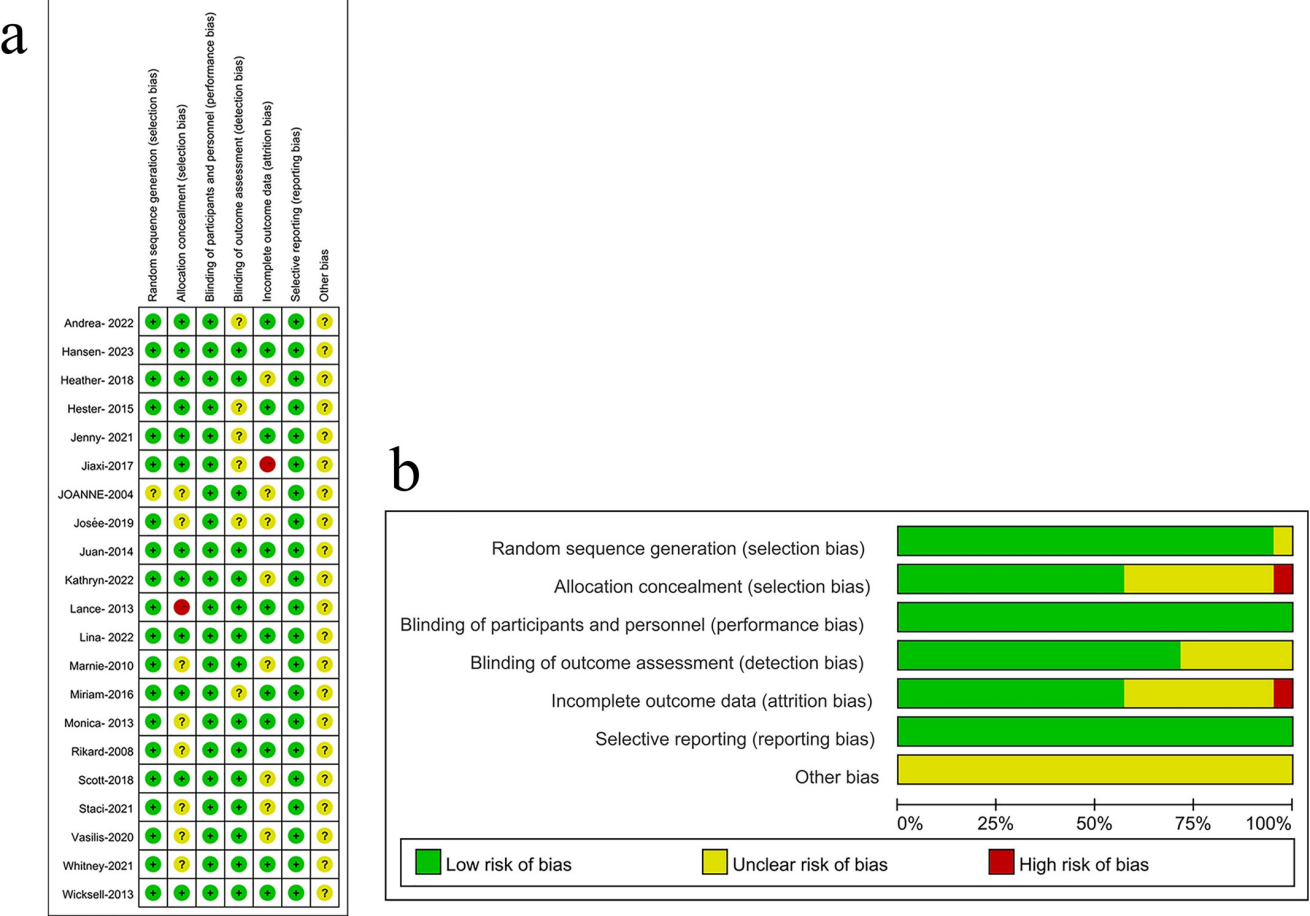


The RCTs included in this meta-analysis all had high methodological quality (Fig 2).

Specifically, all but one of the studies demonstrated random sequence generation [48]. On allocation concealment, one study was assessed as "high risk of bias" [55], eight studies were assessed as "unclear risk of bias" [42, 43, 45, 46, 48, 50, 53, 54]. For performance bias, all studies were considered "low risk of bias." This is because in ACT intervention studies, it is not possible to blind participants or treatment providers. Moreover, no evidence of serious bias was found. For detection bias, six studies did not explicitly indicate whether they blinded the interviewers and were therefore assessed as "some concerns" [47, 54, 56, 57, 59, 60]. In terms of attrition bias, twenty studies were rated as "low risk of bias" [28, 29, 46, 47, 49–51, 53, 55–57, 60]. In these studies, even if there was a loss of some participants, the rate of loss to follow-up and the reasons were similar and comparable between the experimental group and the control; one study was rated as "high risk of bias" [59]; the rest were rated as "unclear risk of bias." For reporting bias, the researchers rated all studies as "low risk of bias" because we can retrieve their registration information or protocols or compare the consistency of methods and results in research. At the same time, the systematic review found no clues about selective reporting. For other biases, all studies were considered "unclear risk of bias" because the authors of these studies were usually professionals in the ACT field.

Fig 2A and 2B show the results of their measurements. Abbreviations of the author names were used in the summary for 21 RCTs.

#### Yates quality assessment tool

Table 1 shows the total score of each study in this assessment.

All RCTs gave the ACT rationale and an adequate description of their intervention content, intervention environment, and duration of treatment. Only nine studies reported adherence to the manual [28, 29, 42, 43, 49–51, 55, 60]. Only two studies reported ACT training specifically for the trial [46, 56]. Thirteen studies documented whether client engagement checks were conducted during the trial [28, 29, 46, 47, 49, 50, 52, 53, 56–60].

### Effect of ACT versus control conditions

This meta-analysis aimed to evaluate the clinical efficacy of ACT compared with control conditions (e.g., WL; TAU). The systematic review performed a subgroup analysis on the follow-up data at three months post-treatment, except for QOL.

#### Effect of post-treatment

**Fig 3:**
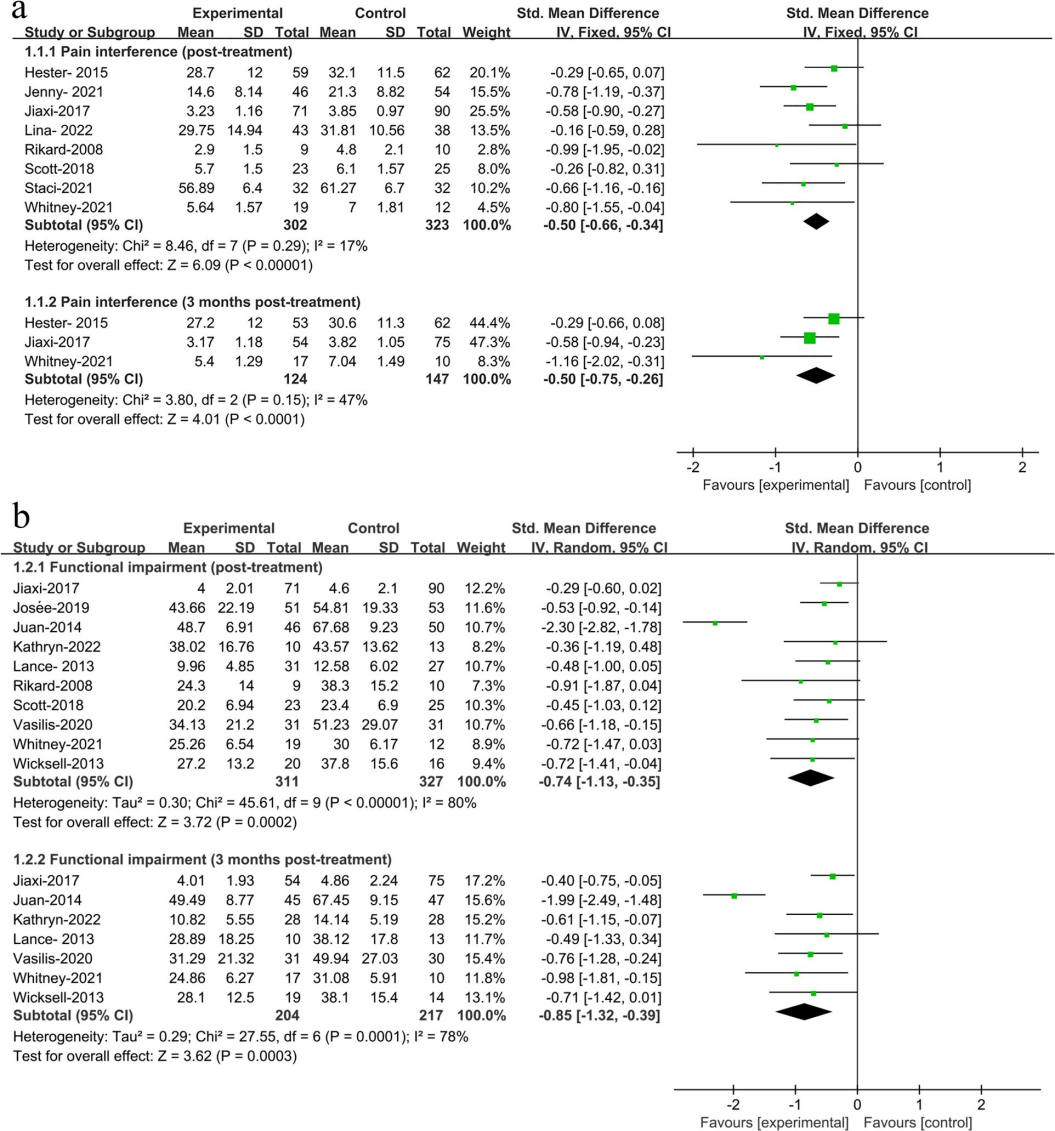


**Fig 4:**
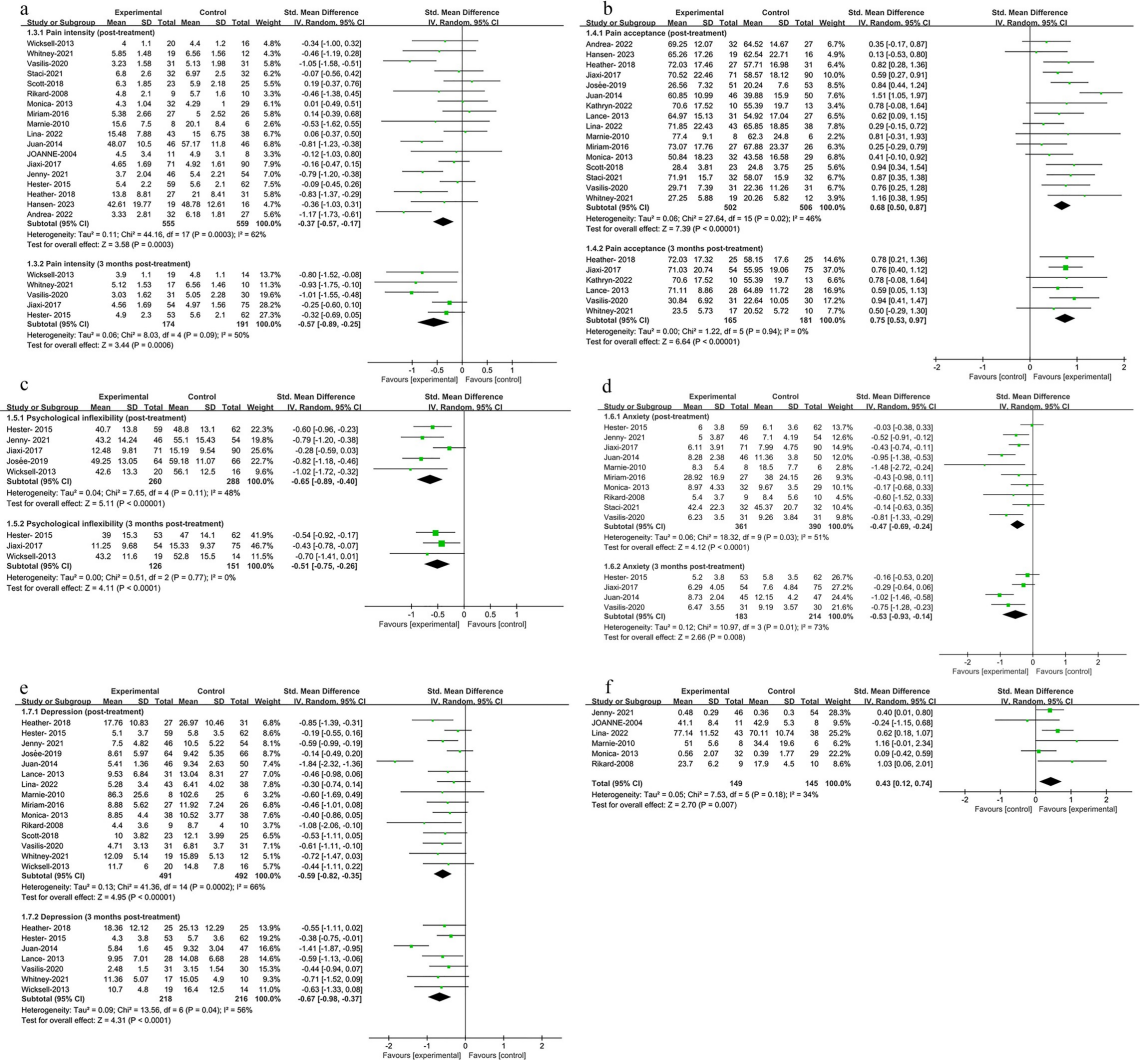


In assessing the impact of ACT post-treatment, our meta-analysis integrated data across eight outcomes, with the results presented in Table 2, Figs 3, and 4. Specifically, we observed the following: Pain Interference: Among 625 participants, there was a significant improvement with an SMD of -0.50 (CI: -0.66 to -0.34, P < 0.001); Functional Impairment: In 638 participants, we noted significant improvements with an SMD of -0.74 (CI: -1.13 to -0.35, P < 0.001); Pain Intensity: Evaluated in 1114 participants, the SMD was -0.37 (CI: -0.57 to -0.17, P < 0.001); Pain Acceptance: Among 1008 participants, the result was positive with an SMD of 0.68 (CI: 0.50 to 0.87, P < 0.001); Psychological Inflexibility: In a group of 548 participants, there was a notable decrease with an SMD of -0.65 (CI: -0.89 to -0.40, P < 0.001); Anxiety: Assessed in 751 participants, the improvement was reflected by an SMD of -0.47 (CI: -0.69 to -0.24, P < 0.001); Depression: Among 983 participants, the analysis showed a significant reduction with an SMD of -0.59 (CI: -0.82 to -0.35, P < 0.001); QOL: In 294 participants, the SMD was 0.43 (CI: 0.12 to 0.74, P < 0.001), favoring ACT.

**Table 2:** Effects of ACT on outcome measures at post-treatment.

Outcome measures	No. studies (significantly favors ACT^a^)	Sample size	SMD^b^ (95% CI^c^)	Statistical heterogeneity
Pain interference	8 (5/8)	625	-0.50 (-0.66, -0.34)*	χ^2^ = 8.46, P < 0.001, *I*^2^ = 17%
Functional impairment	10 (4/10)	638	-0.74 (-1.13, -0.35)*	χ^2^ = 45.61, P < 0.001, *I*^2^ = 80%
Pain intensity	18 (5/18)	1114	-0.37 (-0.57, -0.17)*	χ^2^ = 44.16, P < 0.001, *I*^2^ = 62%
Pain acceptance	16 (9/16)	1008	0.68 (0.50, 0.87)*	χ^2^ = 27.64, P < 0.001, *I*^2^ = 46%
Psychological inflexibility	5 (4/5)	548	-0.65 (-0.89, -0.40)*	χ^2^ = 7.65, P < 0.001, *I*^2^ = 48%
Anxiety	10 (5/10)	751	-0.47 (-0.69, -0.24)*	χ^2^ = 18.32, P < 0.001, *I*^2^ = 51%
Depression	15 (5/15)	983	-0.59 (-0.82, -0.35)*	χ^2^ = 41.36, P < 0.001, *I*^2^ = 66%
Quality of life	6 (3/6)	294	0.43 (0.12, 0.74)*	χ^2^ = 7.53, P < 0.001, *I*^2^ = 17%

^a^ACT indicates acceptance and commitment therapy

^b^SMD, standardized mean difference

^c^CI represents Confidence Interval

I^2^ indicates statistical heterogeneity

*indicated significantly favor ACT intervention

Across these outcomes, ACT consistently showed favorable results. Notably, there was no significant heterogeneity observed in the outcomes of pain interference, psychological inflexibility, and QOL.

#### Effect of three months post-treatment

The meta-analysis focused on data collected three months post-treatment, with results presented in Table 3, Figs 3 and 4. This analysis covered seven specific outcomes, with the following participant numbers and findings: Pain Interference: Analyzed in 251 participants, showing significant improvement with an SMD of -0.50 (CI: -0.75 to -0.26, P < 0.001); Functional Impairment: In 421 participants, a notable improvement was observed with an SMD of -0.85 (CI: -1.32 to -0.39, P < 0.001); Pain Intensity: Evaluated in 365 participants, the SMD was -0.57 (CI: -0.89 to -0.25, P < 0.001); Pain Acceptance: Among 346 participants, the result showed a positive outcome with an SMD of 0.75 (CI: 0.53 to 0.97, P < 0.001); Psychological Inflexibility: With 277 participants, there was a significant reduction with an SMD of -0.51 (CI: -0.75 to -0.26, P < 0.001); Anxiety: Assessed in 397 participants, showing improvement with an SMD of -0.53 (CI: -0.93 to -0.14, P = 0.008); Depression: In 434 participants, the analysis indicated a significant decrease with an SMD of -0.67 (CI: -0.98 to -0.37, P < 0.001).

**Table 3:** Effects of ACT on outcome measures at 3 months post-treatment.

Outcome measures	No. studies (significantly favors ACT^a^)	Sample size	SMD^b^ (95% CI^c^)	Statistical heterogeneity
Pain interference	3 (2/3)	251	-0.50 (-0.75, -0.26)*	χ^2^ = 3.8, P < 0.001, *I*^2^ = 47%
Functional impairment	7 (5/7)	421	-0.85 (-1.32, -0.39)*	χ^2^ = 27.55, P < 0.001, *I*^2^ = 78%
Pain intensity	5 (3/5)	365	-0.57 (-0.89, -0.25)*	χ^2^ = 8.03, P < 0.001, *I*^2^ = 50%
Pain acceptance	6 (4/6)	346	0.75 (0.53, 0.97)*	χ^2^ = 1.22, P < 0.001, *I*^2^ = 0%
Psychological inflexibility	3 (2/3)	277	-0.51 (-0.75, -0.26)*	χ^2^ = 0.51, P < 0.001, *I*^2^ = 0%
Anxiety	4 (2/4)	397	-0.53 (-0.93, -0.14)*	χ^2^ = 10.97, P = 0.008, *I*^2^ = 73%
Depression	7 (3/7)	434	-0.67 (-0.98, -0.37)*	χ^2^ = 13.56, P < 0.001, *I*^2^ = 56%

^a^ACT indicates acceptance and commitment therapy

^b^SMD, standardized mean difference

^c^CI represents Confidence Interval

I^2^ indicates statistical heterogeneity

*indicated significantly favor ACT intervention

These results consistently demonstrated the efficacy of ACT across all outcomes. It’s noteworthy that there was no significant heterogeneity observed in the outcomes of pain interference, pain acceptance, and psychological inflexibility.

### Publication bias

**Fig 5:**
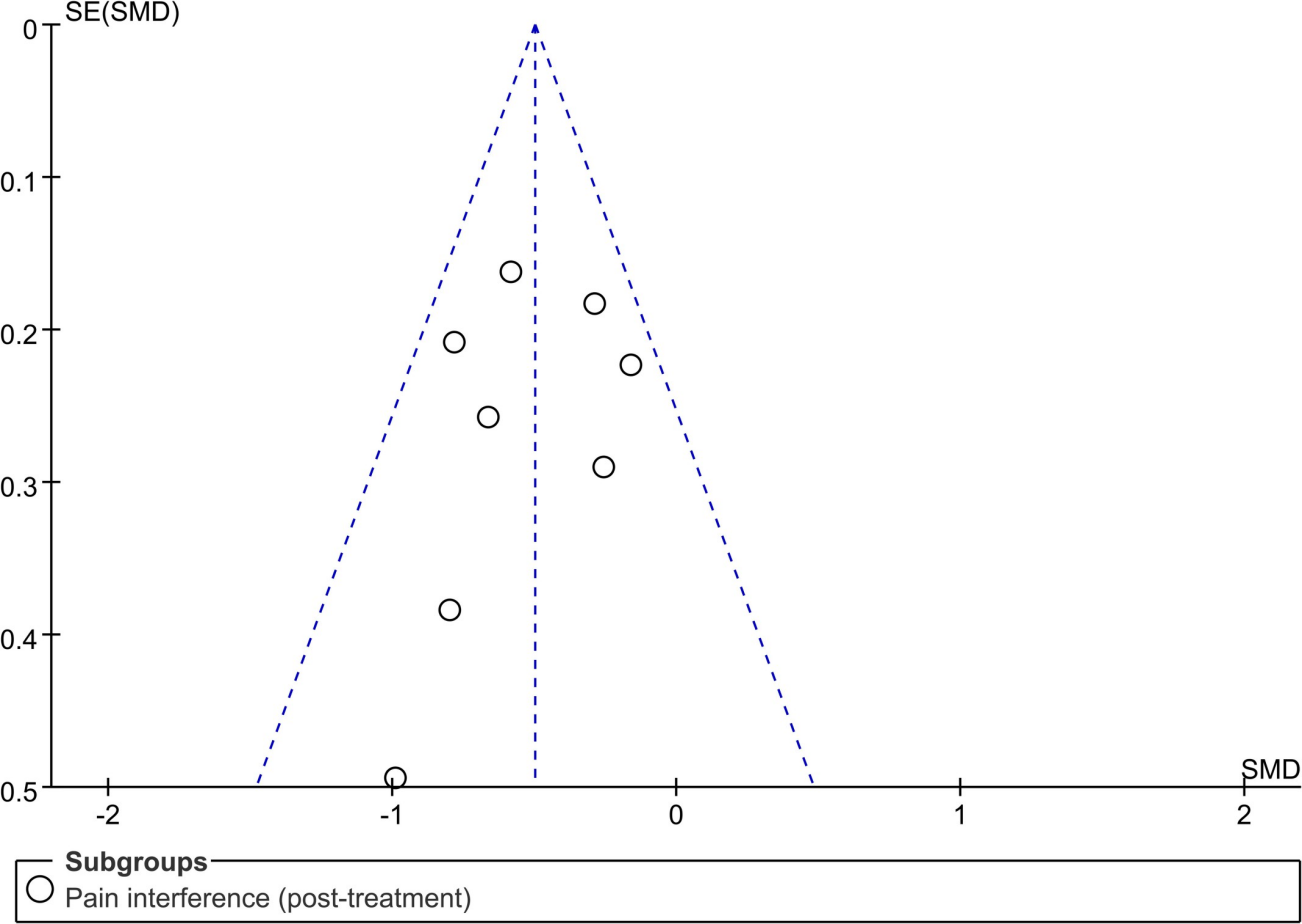


The systematic review plotted funnel plots for the studies that reported pain interference (post-treatment) as an outcome measure to assess publication bias. The results showed that most of the studies were located in the middle and upper part of the funnel plot, and the studies on both sides of the vertical line were roughly symmetrical (Fig 5). Therefore, the systematic review concluded that the risk of publication bias in the current study was low.

### Sensitivity analysis

The systematic review switched between different modes of effect (random effects model and fixed effects model) and found no significant change in the ES. In addition, after each study was excluded in order, the results of the meta-analysis were not significantly changed compared with those before the exclusion, all p < 0.05, which indicates that the results of the meta-analysis were stable.

## Discussion

While some RCTs and meta-analyses support the efficacy of ACT for patients with chronic pain, there remains a need to synthesize evidence from RCTs on ACT interventions in chronic pain for various reasons. Moreover, during this process, we have identified results that diverge from previous studies.

The systematic review included and extracted data from 21 RCTs to examine the effects of ACT on patients with chronic pain. The results showed that at post-treatment and three months post-treatment, ACT had small to large ES on pain interference, functional impairment, pain acceptance, pain intensity, anxiety, depression, psychological inflexibility, and QOL in patients with chronic pain.

For pain interference, ACT was superior to the control group at both post-treatment and three months post-treatment, showing a medium ES. The meta-analysis by Trindade and colleagues reported the same findings [61], which corroborated our meta-analysis results. Both our results and theirs indicated that ACT significantly reduced the pain interference in patients with chronic pain after the intervention and at follow-up, with a change in medium ES.

In the assessment of functional impairment, our study identified moderate to large ES at post-treatment and three months post-treatment for participants who received ACT intervention. This contrasts with the findings of other meta-analyses, such as Hughes’s study, which reported only small ES [25]. The discrepancy in these findings could be attributed to two key factors. Difference in the Number of Studies Included: Our meta-analysis incorporated 10 and 7 studies at two time points for measuring functional impairment, while Hughes’s systematic review included three studies at post-treatment and two studies at three months post-treatment [25]. The inclusion of a greater number of studies in our analysis likely broadened the data spectrum and enhanced statistical power, thereby influencing the estimation of effect sizes and enabling the detection of more significant effects [62]. Variations in Intervention Protocols: Our meta-analysis, incorporating a variety of ACT interventions, stands in contrast to Herbert’s study, which focused solely on technology-supported ACT [26]. This technological approach, while innovative, might limit aspects like personal engagement and therapeutic alliance, potentially reducing efficacy [63]. The absence of direct interaction in technology-supported ACT could lead to less personalized therapy and varied participant-technology dynamics, possibly contributing to the smaller effect sizes observed in such studies [63]. In contrast, our inclusion of diverse ACT protocols, both traditional and technology-supported, provides a broader perspective on ACT’s effectiveness in treating functional impairment, reflecting the complexities of intervention delivery and participant engagement [63].

For pain intensity, the systematic review found that ACT had a small and medium ES improvement for participants at post-treatment and three months post-treatment, respectively. The meta-analysis by Hughes and colleagues reported the same findings [25], and both our results showed significant heterogeneity. After removing the studies one by one and reducing the heterogeneity, our results still showed a small ES. However, Hughes and colleagues only obtained a non-significant result that CI crossed the zero line [25]. The discrepancy between these findings can be attributed to two primary factors. Difference in the Number of Studies Included: Our review included 18 and 5 studies at the respective time points, compared to Hughes’s review, which included only 6 and 4 studies [25]. The larger number of studies in our analysis likely enhanced the statistical power, enabling the detection of a small yet significant effect [62]. Inclusion of Recent High-Quality Research: Our analysis also incorporated studies from the past six years, characterized by higher quality in intervention protocols. Particularly, these recent studies demonstrated improved execution in aspects such as random sequence generation, performance bias, detection bias, and reporting bias. The inclusion of these high-quality studies might have provided a more accurate assessment of ACT’s efficacy, thus impacting the estimation of effect sizes.

For both pain acceptance and psychological inflexibility, our results at both time points showed that ACT was better than the control group, showing a medium ES. Regarding pain acceptance, these two meta-analyses also reported the same findings as ours [25, 61], confirming that ACT can enhance participants’ pain acceptance. Regarding psychological inflexibility, this meta-analysis showed only a small ES [61]. This might be attributed to the study including only RCTs focused on online interventions.

For anxiety, our meta-analysis showed a small and medium ES at two time points, respectively, favoring ACT over the control group. After reducing heterogeneity, the ES was small at both time points, in line with the results of the two meta-analyses [25, 61]. For depression, the systematic review found a medium ES at both time points, supporting the ACT over the control group. This is consistent with the results of the aforementioned meta-analysis [25], which supports the effectiveness of ACT in improving depression.

For QOL, the systematic review showed a small ES favoring ACT over the control group, which was different from the findings of the Hughes and colleagues’ systematic review [25]. This difference can be attributed to two key aspects. Inclusion of More and Recent RCTs: Our review encompassed a larger pool of participants in the RCTs assessing QOL, totaling 294 participants, compared to Hughes’s review, which included only 179 participants [25]. The inclusion of a greater number of recent RCTs enhances the representativeness of our findings and likely brings the results closer to the true values. A larger sample size increases the statistical power of the analysis, potentially leading to more reliable and generalizable findings [62]. Higher Quality of Newer RCTs: The RCTs included in our review are characterized by higher quality, particularly in terms of methodology and execution. Recent studies often adhere to more rigorous standards in research design, such as better randomization processes, effective blinding, and comprehensive reporting. These improvements in study quality contribute to more accurate and trustworthy results, potentially explaining the observed differences in effect sizes for QOL between our study and Hughes’s [25].

According to the Cochrane Risk of Bias assessment [36], most studies included in this meta-analysis demonstrated high-quality evaluations, with a "low risk of bias" rating in random sequence generation, performance bias, detection bias, and reporting bias. However, a closer inspection reveals critical issues, particularly in aspects of allocation concealment and attrition bias. For instance, certain studies lacked explicit and rigorous procedures for allocation concealment [42, 43, 45, 55], failing to provide detailed randomization processes [48] or adequate blinding of researchers and participants [56, 57, 59, 60]. This could potentially introduce selection bias. Similarly, the control over data collection and follow-up phases in some studies was not stringent, leading to incomplete data and raising concerns about the accuracy and reliability of the results [42–44]. These limitations are not to be overlooked in our meta-analysis. Inadequate allocation concealment can lead to overestimation or underestimation of treatment effects [64], while attrition bias could skew results, thereby affecting our overall judgement of the effectiveness of ACT [65]. In synthesizing our findings, we paid particular attention to the potential impact of these issues on our overall conclusions. To add depth and rigor to our conclusions, sensitivity analyses were conducted to assess the extent to which these methodological issues could affect our estimates of effect sizes. This approach not only revealed the range of effect sizes under optimal and worst-case scenarios but also helped us understand the variability of results under different levels of bias.

According to the first part of the Yates Quality Rating Scale [37], the score range for all studies included in this research is between 6 to 8 points, indicating that the trials we incorporated generally possess good quality. This scoring interval suggests that the involved studies have met higher standards in both design and execution. Specifically, these studies exhibited commendable performance in critical quality indicators such as the randomization process, implementation of blinding, selection of control groups, as well as the measurement and reporting of outcomes, thereby providing a solid foundation for the results of our research.

### Strengths and necessity of the study

Over the past decade, several systematic reviews have discussed the effectiveness of clinical staff using ACT interventions for patients with chronic pain. However, this systematic review stands out due to its unique strengths in literature updates, outcome updates, and in-depth discussion of implications and value for practice. Such work is crucial in this field.

Firstly, regarding outcomes, Hughes and team observed that ACT did not lead to significant improvements in QOL for patients with chronic pain [25]. In contrast, our systematic review detected a small ES in favor of ACT. Notably, the systematic review also introduced and delved into a new outcome measure: pain interference. Neither Hughes nor Herbert’s reviews touched upon this metric [25, 26], even though it’s an invaluable measure in pain management. Jensen and associates have argued that beyond pain intensity, a patient’s pain interference score is pivotal for assessing pain severity [32]. Overlooking this metric might result in underwhelming intervention outcomes for chronic pain.

In addition, six years have elapsed since the systematic review by Hughes et al. [25], and numerous new RCTs on the subject have since emerged. This makes it imperative to incorporate these recent studies and refresh the analysis of intervention effects. Our systematic review encompassed 21 RCTs, in contrast to the 11 RCTs in the review by Hughes et al. [25]. We re-analyzed the data, incorporating 10 additional RCTs, and compared our results with those of Hughes and his team. Our analysis showed that, post-intervention, ACT practitioners enhanced the QOL of patients with chronic pain, demonstrating a moderate ES, which diverges from the findings of Hughes et al [25]. Furthermore, our results indicated a significant decrease in psychological inflexibility with a large ES, as opposed to Hughes et al.’s moderate ES. However, in terms of mitigating depression, we noted a small ES, contrasting with Hughes et al.’s moderate ES. Three months post-treatment, we identified a moderate ES in ACT practitioners reducing functional impairment, differing from Hughes et al.’s large ES. On the other hand, our data highlighted a large ES in the reduction of pain intensity by ACT practitioners, while Hughes and his team reported only a moderate ES.

Finally, the systematic review has delved into the implications and relevance of the results from this systematic review specifically for the nursing community, addressing these in our "Implications for Nursing" section. The systematic review ventured into its bearing on nursing education, clinical practice, and pertinent healthcare policies—a dimension not touched upon in prior systematic reviews [23, 25, 26]. Broadly, our systematic review stands distinct in its literature curation, updated findings, and emphasis on nursing relevance.

Generally speaking, this systematic review has unique significance and value in terms of literature screening, results updating, and nursing significance.

### Limitations

Our meta-analysis also has some limitations, mainly in the following aspects. First, it is difficult to eliminate clinical heterogeneity in this meta-analysis, such as different intervention modalities (group versus individual; face-to-face versus online), different intervention durations (two months versus three months), and different clinical populations (ethnicity, age, gender, the type of chronic pain). Although in this meta-analysis, the systematic review used the measurement time of outcome indicators as a subgroup analysis, this can only reduce part of the heterogeneity. In addition, not all of the studies the systematic review included reported all of the outcome measures we needed. This will limit the number of studies included in our quantitative analysis, reducing the accuracy of the results. The language in which we included studies was limited to English, and a search for studies in other languages was lacking. This may have caused us to lose some potential studies that might have been included. Concurrently, our quality assessment, utilizing the Cochrane Risk of Bias Tool and the Yates Quality Rating Scale, revealed inherent limitations and biases inherent to these tools. Subjectivity in assessments and incomplete research reports can distort bias risk evaluations. Although we have endeavored to improve accuracy through assessor training, consistency checks, and sensitivity analyses for publication bias, completely removing tool-induced biases is challenging. Finally, a potential limitation of this study is the absence of preregistration prior to its initiation. The lack of preregistration may lead to reservations among readers regarding the trustworthiness of the research methodology and the interpretation of results, as the absence of a publicly transparent declaration of the research design and analysis plan could be perceived as a source of potential bias. We acknowledge this concern and have endeavored to ensure the transparency and rigor of our research process in other ways. This includes providing detailed descriptions of our literature search strategy, the criteria for study inclusion and exclusion, the data extraction process, and the statistical analysis methods.

### Implications for nursing

Based on our meta-analysis findings, patients with chronic pain benefited from ACT, exhibiting various positive outcomes. This holds several implications for the field of nursing, as detailed below:

ACT in Clinical Nursing Practice: Our findings indicate a moderate enhancement in pain interference, functional impairment, pain acceptance, psychological inflexibility, and depressive symptoms post-treatment, highlighting the significance of incorporating ACT techniques in clinical nursing. To effectively integrate ACT, nurses can include it as part of standard patient education and intervention measures. This could involve educating patients about embracing their pain experiences for pain acceptance, rather than avoiding them. Nurses could introduce mindfulness exercises, such as deep breathing and body scanning, to enhance patients’ body awareness and symptom management. Additionally, employing cognitive defusion techniques in clinical teaching can aid patients in detaching from pain-related thoughts, thereby reducing distress. Importantly, nurses should also focus on assisting patients in identifying and pursuing personal values to enhance life satisfaction and overall quality of life.

Addressing Challenges in Implementing ACT: However, implementing ACT in clinical settings may encounter challenges such as limited resources, time constraints, or the need for specialized training. To overcome these, nurses could utilize brief ACT-based interventions or digital ACT resources, which are time-efficient and easily accessible. Furthermore, regular training programs for nurses in ACT principles and techniques could be beneficial.

Enhanced Follow-up Strategies in ACT-Based Nursing: The sustained improvements in functional impairment and other symptoms at three months post-treatment highlight the importance of effective follow-up in ACT-based nursing. Nurses should develop personalized follow-up plans that include regular reassessments and skill reinforcement sessions to practice ACT techniques. Addressing barriers to continued practice and empowering patients with self-monitoring tools are crucial for maintaining and building upon treatment gains.

### Future research directions in ACT for chronic pain management

Exploring ACT’s Efficacy Across Various Chronic Pain Conditions: Future studies should investigate how ACT specifically benefits different types of chronic pain conditions, such as neuropathic pain, arthritis, or fibromyalgia. Understanding the nuances in ACT’s effectiveness for various pain types can guide clinicians in tailoring interventions to individual patient needs and conditions.

Intervention Delivery by Nursing Staff in Clinical Settings: Current interventions are predominantly led by psychologists. Future studies should explore the feasibility and effectiveness of interventions delivered by nursing staff in hospital settings. This approach could facilitate the integration of ACT into routine patient care and make the therapy more accessible.

Integrating ACT into Nurses’ Routine Care: Future research could explore integrating ACT into the daily interactions between nursing staff and patients, as well as into clinical health education, care planning, and pain management strategies, rather than relying solely on structured courses led by psychologists or on patients’ self-learning. Such integration could enhance patient engagement and make the principles of ACT more applicable to the everyday clinical setting.

Long-Term Follow-up Studies: There is a need for studies with extended follow-up periods, such as one year or more, to observe the long-term effects of ACT on chronic pain. Longer follow-up can provide valuable insights into the sustainability of treatment benefits and patient adherence over time.

Developing Nurse-Led Follow-Up Programs: Future research could focus on creating follow-up programs led by nurses, based on the principles of ACT. These programs could include regular motivational interviews, personalized ACT-based exercises, and continuous monitoring of patients’ progress in specific ACT interventions. Nurses could also provide ongoing support through digital platforms, such as ACT-based mobile applications or online support groups, to facilitate sustained patient engagement and adherence to ACT practices outside the clinical setting.

Controlling Participant Attrition in Research: Future studies should implement strategies to minimize participant dropout rates. High attrition can affect the reliability of results; thus, maintaining participant engagement throughout the study is crucial for obtaining robust and generalizable findings.

## Conclusion

Chronic pain can profoundly influence various facets of an individual’s life. ACT strives to enable patients to participate in numerous meaningful activities, especially when chronic pain is ineliminable, thereby reducing its adverse effects. Our findings demonstrate that ACT exhibited effects ranging from small to large on all outcome measures among patients with chronic pain, and these effects sustained up to three months post-treatment. However, further evidence is still required to ascertain whether these positive impacts persist across specific pain conditions and more extended periods. We hope that future RCTs will be conducted in more specific chronic pain settings with longer follow-ups to draw more precise and relevant conclusions.

## Supporting information

S1 Text Checklist of items to include when reporting a systematic review or meta-analysis. (DOC)

S1 Raw data (XLSX)

S1 Appendix (DOCX)
