# Supplementary material for: Acceptance and commitment therapy for patients with chronic pain: A systematic review and meta-analysis on psychological outcomes and quality of life
Source: PLoS One. 2024 Jun 14;19(6):e0301226. doi: 10.1371/journal.pone.0301226 (PMC11178235; doi:10.1371/journal.pone.0301226)
Supplement: S1 Text — (DOC) [file pone.0301226.s001.doc]

Text S1 - Checklist of items to include when reporting a systematic review or meta-analysis

| Section/topic | # | Checklist item | Reported on page # |
| --- | --- | --- | --- |
| **TITLE** | | | |
| Title | 1 | Identify the report as a systematic review, meta-analysis, or both. | Line 3, page 2; |
| **ABSTRACT** | | | |
| Structured summary | 2 | Provide a structured summary including, as applicable: background; objectives; data sources; study eligibility criteria, participants, and interventions; study appraisal and synthesis methods; results; limitations; conclusions and implications of key findings; systematic review registration number. | Line 28 - 54,  page 2 - 3; |
| **INTRODUCTION** | | | |
| Rationale | 3 | Describe the rationale for the review in the context of what is already known. | Line 95 - 147,  page 5 - 7; |
| Objectives | 4 | Provide an explicit statement of questions being addressed with reference to participants, interventions, comparisons, outcomes, and study design (PICOS). | Line 149 - 164,  page 7 - 8; |
| **METHODS** | | | |
| Protocol and registration | 5 | Indicate if a review protocol exists, if and where it can be accessed (e.g., Web address), and, if available, provide registration information including registration number. | NONE |
| Eligibility criteria | 6 | Specify study characteristics (e.g., PICOS, length of follow-up) and report characteristics (e.g., years considered, language, publication status) used as criteria for eligibility, giving rationale. | Line 180 - 202,  page 9 - 10; |
| Information sources | 7 | Describe all information sources (e.g., databases with dates of coverage, contact with study authors to identify additional studies) in the search and date last searched. | Line 170 - 178,  page 8 - 9; |
| Search | 8 | Present full electronic search strategy for at least one database, including any limits used, such that it could be repeated. | Line 864 - 974,  page 37 - 40; |
| Study selection | 9 | State the process for selecting studies (i.e., screening, eligibility, included in systematic review, and, if applicable, included in the meta-analysis). | Line 204 - 221,  page 10 - 11; |
| Data collection process | 10 | Describe method of data extraction from reports (e.g., piloted forms, independently, in duplicate) and any processes for obtaining and confirming data from investigators. | Line 223 - 234,  page 11; |
| Data items | 11 | List and define all variables for which data were sought (e.g., PICOS, funding sources) and any assumptions and simplifications made. | Line 180 - 202,  page 9 - 10; |
| Risk of bias in individual studies | 12 | Describe methods used for assessing risk of bias of individual studies (including specification of whether this was done at the study or outcome level), and how this information is to be used in any data synthesis. | Line 236 - 255,  page 11 - 12; |
| Summary measures | 13 | State the principal summary measures (e.g., risk ratio, difference in means). | Line 285 - 292,  page 14; |
| Synthesis of results | 14 | Describe the methods of handling data and combining results of studies, if done, including measures of consistency (e.g., I^2^) for each meta-analysis. | Line 257 - 292,  page 12 - 14; |
| Risk of bias across studies | 15 | Specify any assessment of risk of bias that may affect the cumulative evidence (e.g., publication bias, selective reporting within studies). | Line 291,  page 14; |
| Additional analyses | 16 | Describe methods of additional analyses (e.g., sensitivity or subgroup analyses, meta-regression), if done, indicating which were pre-specified. | Line 268 - 284,  page 13 - 14; |
| RESULTS | | | |
| Study selection | 17 | Give numbers of studies screened, assessed for eligibility, and included in the review, with reasons for exclusions at each stage, ideally with a flow diagram. | Line 295 - 302,  page 14 - 15; |
| Study characteristics | 18 | For each study, present characteristics for which data were extracted (e.g., study size, PICOS, follow-up period) and provide the citations. | Line 302 - 311,  page 14 - 15;  Table 1; |
| Risk of bias within studies | 19 | Present data on risk of bias of each study and, if available, any outcome-level assessment (see Item 12). | Line 313 - 344,  page 15 - 16; |
| Results of individual studies | 20 | For all outcomes considered (benefits or harms), present, for each study: (a) simple summary data for each intervention group and (b) effect estimates and confidence intervals, ideally with a forest plot. | Line 346 - 393,  page 16 - 19; |
| Synthesis of results | 21 | Present results of each meta-analysis done, including confidence intervals and measures of consistency. | Line 346 - 393,  page 16 - 19; |
| Risk of bias across studies | 22 | Present results of any assessment of risk of bias across studies (see Item 15). | Line 313 - 344,  page 15 - 16; |
| Additional analysis | 23 | Give results of additional analyses, if done (e.g., sensitivity or subgroup analyses, meta-regression [see Item 16]). | Line 377 - 415,  page 18 - 20; |
| DISCUSSION | | | |
| Summary of evidence | 24 | Summarize the main findings including the strength of evidence for each main outcome; consider their relevance to key groups (e.g., health care providers, users, and policy makers). | Line 427 - 499,  page 21 - 24; |
| Limitations | 25 | Discuss limitations at study and outcome level (e.g., risk of bias), and at review level (e.g., incomplete retrieval of identified research, reporting bias). | Line 569 - 587,  page 27 - 28; |
| Conclusions | 26 | Provide a general interpretation of the results in the context of other evidence, and implications for future research. | Line 653 - 662,  page 31 - 32; |
| FUNDING | | | |
| Funding | 27 | Describe sources of funding for the systematic review and other support (e.g., supply of data); role of funders for the systematic review. | Line 664 - 671,  page 32; |
