# Supplementary material for: Acceptance and commitment therapy for patients with chronic pain: A systematic review and meta-analysis on psychological outcomes and quality of life
Source: PLoS One. 2024 Jun 14;19(6):e0301226. doi: 10.1371/journal.pone.0301226 (PMC11178235; doi:10.1371/journal.pone.0301226)
Supplement: S1 Appendix — (DOCX) [file pone.0301226.s003.docx]

**Appendix A**

**Search strategies in Pubmed**

(((((Acceptance[Title/Abstract] or commitment[Title/Abstract]) and (therap*[Title/Abstract] or strateg*[Title/Abstract] or treatment*[Title/Abstract] or intervention*[Title/Abstract] or technique*[Title/Abstract])) OR ("Acceptance and commitment therapy"[Title/Abstract] or ACT[Title/Abstract])) OR ("Acceptance and Commitment Therapy"[MeSH])) AND ((((sciatica [Title/Abstract] or lumbago [Title/Abstract] or fibromyalg* [Title/Abstract] or (diabet* [Title/Abstract] and neuropath* [Title/Abstract]) or (sudeck* [Title/Abstract] and atroph* [Title/Abstract]) or causalg* [Title/Abstract] or whiplash [Title/Abstract] or "whip lash" [Title/Abstract] or polymyalg* [Title/Abstract] or ("failed back" [Title/Abstract] and surg* [Title/Abstract])) OR ((neuralg* [Title/Abstract]) AND (chronic [Title/Abstract] or trigemin* [Title/Abstract] or herp* [Title/Abstract]))) OR (((chronic* [Title/Abstract] or back [Title/Abstract] or musculoskel* [Title/Abstract] or intractabl* [Title/Abstract] or neuropath* [Title/Abstract] or "phantom limb" [Title/Abstract] or neck [Title/Abstract] or myofasc* [Title/Abstract] or "Temporomandibular Joint*" [Title/Abstract] or "temperomandibular joint*" [Title/Abstract] or "tempromandibular joint*" [Title/Abstract] or central [Title/Abstract] or "post stroke" [Title/Abstract] or poststroke [Title/Abstract] or complex [Title/Abstract] or regional [Title/Abstract] or "spinal cord" [Title/Abstract]) and (pain* [Title/Abstract] or ache* [Title/Abstract])))) OR (Pain[MeSH]))) AND (("Randomized Controlled Trial" [Publication Type] OR "Controlled Clinical Trial" [Publication Type] OR "Clinical Trials as Topic"[Mesh:NoExp] OR randomized[Title/Abstract] OR placebo [Title/Abstract] OR randomly[Title/Abstract] OR trial[Title/Abstract]) NOT ("Animals"[Mesh] NOT "Humans"[Mesh]))

**Search strategies in Embase**

‘crossover procedure':de OR 'double-blind procedure':de OR ‘randomized controlled trial':de OR ‘single-blind procedure':de OR (random* OR factorial* OR crossover* OR cross NEXT/1 over* OR placebo* OR doubl* NEAR/1 blind* OR singl* NEAR/1 blind* OR assign* OR allocat* OR volunteer*):de,ab,ti

AND

(((("Pain"[Mesh]) OR ((pain* [Title/Abstract] or ache* [Title/Abstract]) AND (chronic* [Title/Abstract] or back [Title/Abstract] or musculoskel* [Title/Abstract] or intractabl* [Title/Abstract] or neuropath* [Title/Abstract] or "phantom limb" [Title/Abstract] or neck [Title/Abstract] or myofasc* [Title/Abstract] or "Temporomandibular Joint*" [Title/Abstract] or "temperomandibular joint*" [Title/Abstract] or "tempromandibular joint*" [Title/Abstract] or central [Title/Abstract] or "post stroke" [Title/Abstract] or poststroke [Title/Abstract] or complex [Title/Abstract] or regional [Title/Abstract] or "spinal cord" [Title/Abstract]))) OR (((("failed back" [Title/Abstract] and surg* [Title/Abstract]) OR (sudeck* [Title/Abstract] and atroph* [Title/Abstract])) OR (diabet* [Title/Abstract] and neuropath* [Title/Abstract])) OR ((neuralg*) AND (chronic [Title/Abstract] or trigemin* [Title/Abstract] or herp* [Title/Abstract]))))

AND

("Acceptance and Commitment Therapy"/exp)

**Search strategies in PsycINFO/ CINAHL (EBSCO)**

((((Any Field: ("Acceptance and Commitment Therapy")))) OR (((Any Field: (Acceptance)) OR (Any Field: (commitment))) AND ((Any Field: (therap*)) OR (Any Field: (strateg*)) OR (Any Field: (treatment*)) OR (Any Field: (intervention*)) OR (Any Field: (technique*)))) OR (((MeSH: ("acceptance and commitment therapy"))))) AND ((((MeSH: (pain)))) OR (((Any Field: (sciatica)) OR (Any Field: (lumbago)) OR (Any Field: (fibromyalg*)) OR (((Any Field: (chronic)) OR (Any Field: (trigemin*)) OR (Any Field: (herp*))) AND (Any Field: (neuralg*))) OR ((Any Field: (diabet*)) AND (Any Field: (neuropath*))) OR ((Any Field: (sudeck*)) AND (Any Field: (atroph*))) OR (Any Field: (causalg*)) OR (Any Field: (whiplash)) OR (Any Field: ("whip lash")) OR (Any Field: (polymyalg*)) OR ((Any Field: ("failed back")) AND (Any Field: (surg*)))) OR (((((Any Field: (pain*))) OR ((Any Field: (ache*))))) AND ((((Any Field: (chronic*))) OR ((Any Field: (back))) OR ((Any Field: (musculoskel*))) OR ((Any Field: (intractabl*))) OR ((Any Field: (neuropath*))) OR ((Any Field: ("phantom limb"))) OR ((Any Field: ("fantom limb"))) OR ((Any Field: (neck))) OR ((Any Field: (myofasc*))) OR ((Any Field: ("Temporomandibular Joint*"))) OR ((Any Field: ("temperomandibular joint*"))) OR ((Any Field: ("tempromandibular joint*"))) OR ((Any Field: (central))) OR ((Any Field: ("post stroke"))) OR ((Any Field: (poststroke))) OR ((Any Field: (complex))) OR ((Any Field: (regional))) OR ((Any Field: ("spinal cord")))))))

**Search strategies in the Cochrane Library**

#1: chronic* OR back OR musculoskel* OR intractabl* OR neuropath* OR “phantom limb” OR “fantom limb” OR neck OR myofasc* OR “Temporomandibular Joint*” OR “temperomandibular joint*” OR “tempromandibular joint*” OR central OR “post stroke” OR poststroke OR complex OR regional OR “spinal cord” OR (sciatica or lumbago or fibromyalg* or ((chronic or trigemin* or herp*) and neuralg*) or (diabet* and neuropath*) or (sudeck* and atroph*) or causalg* or whiplash or “whip lash” or polymyalg* or (“failed back” and surg*) OR sciatica OR lumbago OR fibromyalg* OR causalg* OR whiplash OR “whip lash” OR polymyalg*

#2: pain* OR ache*

#3: #1 AND #2

#4: (chronic OR trigemin* OR herp*):ti,ab,kw

#5: (neuralg*):ti,ab,kw

#7: #4 AND #5

#8 (ACT adj6 therap*).ti,ab OR (acceptance adj6 therap*).ti,ab OR (acceptance adj6 strateg*).ti,ab OR (acceptance adj6 treatment*).ti,ab OR (acceptance adj6 intervention*).ti,ab OR (acceptance adj6 technique*).ti,ab OR ((Acceptance:ab,ti or commitment:ab,ti) and (therap*:ab,ti or strateg*:ab,ti or treatment*:ab,ti or intervention*:ab,ti or technique*:ab,ti) :ab,ti) OR ((Acceptance or commitment) and (therap* or strateg* or treatment* or intervention* or technique*))

#9 #7 AND #8

**Search strategies in Web of Science**

((TI=(Acceptance OR commitment) OR AB=(Acceptance OR commitment)) AND (TI=(therap* OR strateg* OR treatment* OR intervention* OR technique*) OR AB=(therap* OR strateg* OR treatment* OR intervention* OR technique*))) OR (TI=("Acceptance and commitment therapy" OR ACT) OR AB=("Acceptance and commitment therapy" OR ACT)) OR TS=("Acceptance and Commitment Therapy") AND ((TI=(sciatica OR lumbago OR fibromyalg*) OR AB=(sciatica OR lumbago OR fibromyalg*) OR (TI=(diabet*) AND TI=(neuropath*)) OR AB=(diabet* AND neuropath*) OR (TI=(sudeck*) AND TI=(atroph*)) OR AB=(sudeck* AND atroph*) OR TI=(causalg* OR whiplash OR "whip lash" OR polymyalg* OR "failed back") OR AB=(causalg* OR whiplash OR "whip lash" OR polymyalg* OR "failed back")) OR (TI=(neuralg*) AND (TI=(chronic OR trigemin* OR herp*) OR AB=(chronic OR trigemin* OR herp*))) OR ((TI=(chronic* OR back OR musculoskel* OR intractabl* OR neuropath* OR "phantom limb" OR neck OR myofasc* OR "Temporomandibular Joint*" OR "temperomandibular joint*" OR "tempromandibular joint*" OR central OR "post stroke" OR poststroke OR complex OR regional OR "spinal cord") OR AB=(chronic* OR back OR musculoskel* OR intractabl* OR neuropath* OR "phantom limb" OR neck OR myofasc* OR "Temporomandibular Joint*" OR "temperomandibular joint*" OR "tempromandibular joint*" OR central OR "post stroke" OR poststroke OR complex OR regional OR "spinal cord")) AND (TI=(pain* OR ache*) OR AB=(pain* OR ache*))))
